# Prevalence and determinants of maternal near miss in Ethiopia: a systematic review and meta-analysis, 2015–2023

**DOI:** 10.1186/s12905-023-02523-9

**Published:** 2023-07-19

**Authors:** Abraham Negash, Addisu Sertsu, Dechasa Adare Mengistu, Aklilu Tamire, Adisu Birhanu Weldesenbet, Mesay Dechasa, Kabtamu Nigussie, Tilahun Bete, Elias Yadeta, Tegenu Balcha, Gebiso Roba Debele, Deribe Bekele Dechasa, Hamdi Fekredin, Habtamu Geremew, Jerman Dereje, Fikadu Tolesa, Magarsa Lami

**Affiliations:** 1 School of Nursing and Midwifery, College of Health and Medical Sciences, Haramaya University, Harar, Ethiopia; 2 School of Environmental Health, College of Health and Medical Sciences, Haramaya University, Harar, Ethiopia; 3 School of Public Health, College of Health and Medical Sciences, Haramaya University, Harar, Ethiopia; 4 School of Pharmacy, College of Health and Medical Sciences, Haramaya University, Harar, Ethiopia; 5 College of Health Sciences, Mattu University, Mattu, Ethiopia; 6 College of Health Sciences, Oda Bultum University, Chiro, Ethiopia; 7 College of Health Sciences, Salale University, Fitche, Ethiopia

## Abstract

**Background:**

One of the most challenging problems in developing countries including Ethiopia is improving maternal health. About 303,000 mothers die globally, and one in every 180 is at risk from maternal causes. Developing regions account for 99% of maternal deaths. Maternal near miss (MNM) resulted in long-term consequences. A systematic review and meta-analysis was performed to assess the prevalence and predictors of maternal near miss in Ethiopia from January 2015 to March 2023.

**Methods:**

A systematic review and meta-analysis cover both published and unpublished studies from different databases (PubMed, CINHAL, Scopus, Science Direct, and the Cochrane Library) to search for published studies whilst searches for unpublished studies were conducted using Google Scholar and Google searches. Preferred Reporting Items for Systematic Reviews and Meta-Analyses (PRISMA) guidelines were used. Duplicated studies were removed using Endnote X8. The paper quality was also assessed based on the JBI checklist. Finally, 21 studies were included in the study. Data synthesis and statistical analysis were conducted using STATA Version 17 software. Forest plots were used to present the pooled prevalence using the random effect model. Heterogeneity and publication bias was evaluated using Cochran’s Q test, (Q) and I squared test (I^2^). Subgroup analysis based on study region and year of publication was performed.

**Result:**

From a total of 705 obtained studies, twenty-one studies involving 701,997 pregnant or postpartum mothers were included in the final analysis. The national pooled prevalence of MNM in Ethiopia was 140/1000 [95% CI: 80, 190]. Lack of formal education [AOR = 2.10, 95% CI: 1.09, 3.10], Lack of antenatal care [AOR = 2.18, 95% CI: 1.33, 3.03], history of cesarean section [AOR = 4.07, 95% CI: 2.91, 5.24], anemia [AOR = 4.86, 95% CI: 3.24, 6.47], and having chronic medical disorder [AOR = 2.41, 95% CI: 1.53, 3.29] were among the predictors of maternal near misses from the pooled estimate.

**Conclusion:**

The national prevalence of maternal near miss was still substantial. Antenatal care is found to be protective against maternal near miss. Emphasizing antenatal care to prevent anemia and modifying other chronic medical conditions is recommended as prevention strategies. Avoiding primary cesarean section is recommended unless a clear indication is present. Finally, the country should place more emphasis on strategies for reducing MNM and its consequences, with the hope of improving women's health.

**Supplementary Information:**

The online version contains supplementary material available at 10.1186/s12905-023-02523-9.

## Background

Globally, 303 000 mothers die each year from maternal causes, with one in every 180 at risk; developing regions account for 99% of maternal deaths [1]. Millions of women suffered from pregnancy and its complications, including death, and the majority (94%) of these deaths occurred in low- and middle-income countries [2]. Sub-Saharan African countries continue to share the largest portion of maternal mortality [3]; about 66% of global MMR accounts for sub-Saharan Africa alone [4]. However, it is rare in absolute numbers in the community, possibly due to underreporting by healthcare providers and managers [5].

Direct obstetric causes like hemorrhage, hypertensive disorder of pregnancy (pre-eclampsia, eclampsia), postpartum sepsis, obstructed labor, uterine rupture, and abortion-related death are among the most common causes of maternal morbidity and mortality [2, 6, 7].

For every woman who dies from pregnancy or childbirth-related causes, about twenty more experienced maternal near miss [8]. World Health Organization (WHO) defines a maternal near-miss (MNM) as a woman who nearly died but survived a complication that occurred during pregnancy, childbirth, or within 42 days of termination of pregnancy [9, 10].

The national annual incidence of maternal near miss was 7.2 per 1,000 live births in Kenya [11], 22.1 per 1000 live births in Sudan [12], and 23.6 per 1,000 live births in Tanzania [13]. In Uganda, the MNM rate was 287.7/1000 pregnancies [14]. Maternal near miss in Ethiopia ranges from 4.97% to 29.7% [15, 16], one study reported a 50.4 per 1000 live births ratio [17].

Studying MNM is very important, as maternal mortality is more likely to be underreported by healthcare providers and managers, especially in low-income countries [18]. MNM shows the quality of obstetric intervention to save the lives of mothers and also provides the chance of interviewing the mother [19].

All countries must contribute to achieve the global target of MMR of less than 70 per 1,000 live births, and no country should have MMR of more than 140 per 1,000 live births by 2030 [20, 21]. Ethiopia is one of the countries that strive to reduce maternal mortality through different strategies, such as the promotion of maternal health, the provision of free maternity services and the provision of supplies [22]; however maternal mortality remains a public concern [10, 23, 24].

MNM is associated with life-threatening pregnancy outcomes like stillbirth, birth asphyxia, and low birth weight [25–28]. This bad pregnancy outcome can be reduced by identifying predictors, implementing intervention for context-specific quality improvement [29], and addressing modifiable risk factors accordingly [30].

Different predictors that were associated with maternal near misses in Ethiopia include, delay to seek health care, referred from the health facility, history of cesarean Section. [31–33], no formal education [28, 32], travelling greater than 60 min to reach the place of final care [15, 32, 33], induction of labor [15, 32, 33], age less than 16 [32], history of chronic medical disorders [28, 32, 33], lack of ANC [15, 33], lack of birth preparedness and complication readiness plan [33], less monthly income, and rural residence [28].

Understanding the estimated national burden of MNM and its predictors is very important. This will enable policymakers and anyone else who wants to take action to reduce maternal morbidity and mortality to do so based on evidence. Apart from different studies across the countries that are inconsistent and make generalizability difficult, there is a paucity of pooled national evidence on the burden of MNM and its predictors. Therefore, this study aimed to determine the pooled prevalence of maternal near-miss and its determinants among women in Ethiopia by including a study published after 2015, as it was a turning point from millennium development goals to sustainable development goals.

## Methods

### Systematic review registration and reporting of findings

The protocol for this study has been registered on the International Prospective Register of Systematic Review (PROSPERO), the University of York Center for Reviews and Dissemination (https://www.crd.york.ac.uk/) with registration number CRD42023393803. The finding of the study was reported according to the Preferred Reporting Items for Systematic Reviews and Meta-Analysis (PRISMA 2020) [34] guidelines.

### Eligibility criteria and study selection

The systematic review and meta-analysis were designed to determine the magnitude of MNM and its determinants among pregnant and postpartum women in Ethiopia. A maternal near miss is a woman who nearly died but survived a complication that occurred during pregnancy, childbirth or within 42 days of termination of pregnancy [10].

This study included all published studies (cross-sectional, case–control, and cohort) that reported the magnitude and/or determinants of a maternal near-miss in Ethiopia, with full text and written in English. However, studies that only included abstracts, case reports and written in languages other than English were excluded. End note X8 was used to remove the duplicated result. Three authors (AN, ML, TB), screened the article by reading the title and abstract based on predefined inclusion and exclusion criteria.

### Sources and search strategy

The systematic review took into account English-language studies that were published between January 2015 and March 2023. Searches for published research were conducted using electronic databases like PubMed/Medline, Scopus, CINHAL, Science Direct, and the Cochrane Library, whilst searches for unpublished studies were conducted using Google Scholar, Advanced Google Search and Google. Keywords and medical subject heading terms were used to search an electronic data repository. The discovered PECO components were connected using Boolean operators.

The search strategy for advanced PubMed includes: *((((("epidemiology"[Subheading] OR "epidemiology"[All Fields] OR "prevalence"[All Fields] OR "prevalence"[MeSH Terms]) OR determinants[All Fields]) OR burden[All Fields]) AND ("mothers"[MeSH Terms] OR "mothers"[All Fields] OR "maternal"[All Fields]) AND "near miss"[All Fields]) AND (("mothers"[MeSH Terms] OR "mothers"[All Fields] OR "maternal"[All Fields]) AND near[All Fields] AND miss[All Fields])) AND Ethiopia[Title].*

### For scopus search

Aditional searching (ALL (prevalence) AND ALL(maternal AND nearmiss) OR TITLE-ABS-KEY ( maternal AND near-miss) AND TITLE-ABS-KEY ( predictors) OR ALL (associated AND factors) AND TITLE-ABS-KEY (Ethiopia)) were used. Then all identified keywords and index terms were checked across all databases. Finally the reference lists of all identified articles were searched for further articles.

### Quality assessment and data extraction

Data extraction was done by five authors (ML, AN, AS, HD and FT) by using an extraction format prepared on Microsoft Excel. The extracted data were first author name, publication year, publication place, study design, sample size, sampling method, maternal near miss, determinants with odd ratio, and causes that resulted in maternal near miss.

Each screened article was evaluated by five authors (AN, ML, AS, AB, and DA) for quality assurance using standardized critical evaluation tools, Joanna Briggs Institute (JBI) Critical Appraisal tools [35]. Then the quality of each included article was classified as high (80% or above), moderate (65%–80%), or low (less than 65%). The final inclusion was determined by reading the full text of the articles by five authors (AS, EY, KN, Til. B and AT). Finally, the included article was verified by (MD, DA, HF and GD). Any disagreement was resolved by the discussion after the same procedure was repeated.

### Outcome of interest

The primary outcome of this systematic review is the magnitude of maternal near miss in Ethiopia. The secondary outcome is determinants of maternal near miss.

### Statistical analysis and publication bias

Data synthesis and statistical analysis were conducted using STATA version 17. The random effect model of analysis was used as a method of meta-analysis to reduce the heterogeneity of included studies. Subgroup meta-analysis was done by study setting, sample size and year of publication. A forest plot was used to present the result of the meta-analysis. Heterogeneity among included articles was evaluated using Cochran’s Q test, (Q) and I squared test (I^2^). I^2^ test statistics of 25%, 50% and 75% were declared as low, moderate and high heterogeneity, respectively. The effect of factors associated with maternal near misses was pooled. The investigators checked for potential publication bias through visual inspection of a funnel plot and Egger’s Regression Test. A p-value of less than 0.05 was used as statistical evidence of publication bias. Finally, the findings of the included studies were first presented using a narrative synthesis and followed by a meta-analysis chart.

## Result

### Study identification

**Fig. 1:**
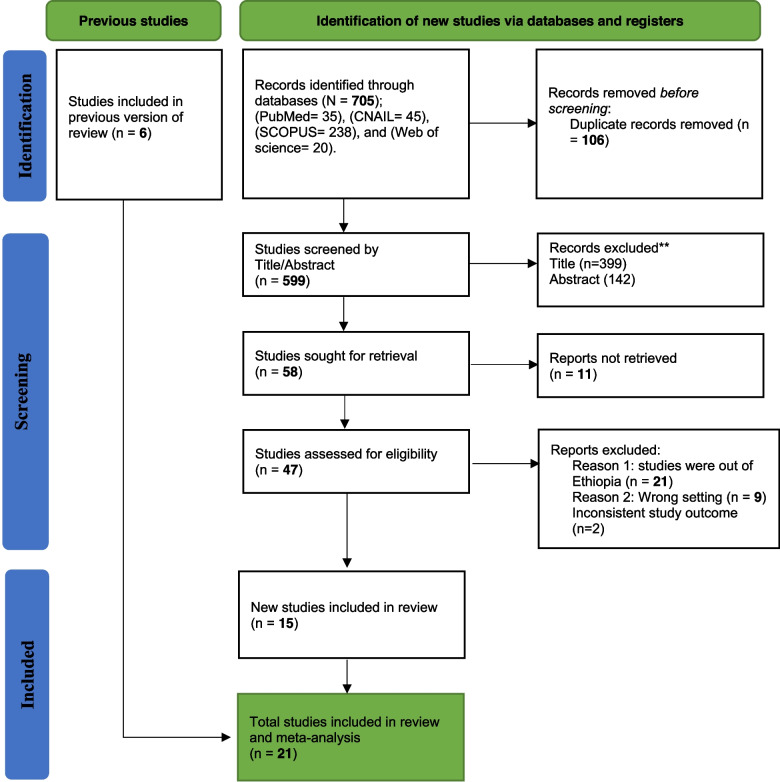
PRISMA flow diagram of the studies included in final systematic review and meta-analysis of maternal near miss in Ethiopia, 2023 [36]

Initially, 705 studies were retrieved from electronic databases like PubMed/Midline, Scopus, CINHAL and Science Direct. Unpublished studies were searched from Google Scholar and advanced Google search and Google. The major reason for exclusion was duplication and mismatch. 105 duplicate was found and removed. 599 studies were screened by title and abstract 541 of them were removed due to mismatch. 58 studies were sought for retrieval and 11 were not retrieved. A full text was assessed for illegibility (English language, report prevalence of MNM and/or its predictors, present in full text, a study done in Ethiopia after 2015 and moderate and above in quality) and finally, 21 studies were included in a systematic review and meta-analysis (Fig. 1).

### Study characteristics

A total of 701,997 pregnant or postpartum mothers who visited the health facility for Obstetrics or Gynecologic services from 21 studies were included. A study published from January 2015 to March 2023 in Ethiopia, of which 5 studies were from the Amhara region, 5 from the Oromia region, 4 studies were from the Southern Nation Nationalities and Peoples (SNNP) region, 1 study from the Harar region, 2 studies from the Tigray region, 3 studies from Addis Abeba city, and another study that was done nationally, were included. Eight of them were published before 2020, while the rest were published after 2020. Regarding the quality of included studies 10 were moderate while the rest 11 were high quality based on JBI (Additional file 1). 12 of 22 studies used a cross-sectional study design, while the rest used a case–control, except for one study that used a survey from national data. From the total included studies 8 used record review for data collection, while 12 studies used interviews supported by chart review. The sample size ranges from 296 to 78,195 (Table 1).

**Table 1:** Characteristics of studies included in systematic review and meta-analysis of maternal near miss in Ethiopia, 2023

Author (year of publication)	Study setting	Study design	Sample size	No of MNM	Prevalence (%)	Data collection method
Tenaw et al. (2021) [37]	Harar	Cross-sectional	1211	108	8.9	Record review
Mekonnen et al. (2021) [38]	Oromia	Cross-sectional	296	85	28.7	Record review
Teka et al. (2022) [39]	Tigray	Cross-sectional	5116	146	2.9	Record review
Gebremariam et al. (2022) [40]	Amhara	Cross-sectional	905	129	14.3	Record review
Kumela et al. (2020) [15]	Oromia	Case–control	1227	61	4.9	Interview and record review
Geleto et al. (2020) [41]	National	Survey	323,824	67,567	20.8	National data set from EPHI
Liyew et al. (2017) [22]	Addis Ababa	Cross-sectional	29,697	238	0.8	Record review
Wakgar et al. (2019) [42]	SNNP	Cross-sectional	15,059	501	3.3	Record review
Yemaneh et al. (2020) [43]	SNNP	Cross-sectional	845	210	24.8	Interview and record review
Woldeyes et al. (2018) [17]	Oromia	Cross-sectional	2737	138	5.0	Interview and record review
Worke et al. (2019) [44]	Amhara	Cross-sectional	572	152	26.6	Interview and record review
Asaye et al. (2020) [45]	Amhara	Cross-sectional	303	48	15.8	Interview and record review
Dile et al. (2015) [46]	Amhara	Cross-sectional	806	188	23.3	Interview and concealed observation
Teshome et al. (2022) [47]	Amahara	Case–control	264	88	N/A	Interview and record review
Danusa et a. (2022) [48]	Oromia	Case–control	664	166	N/A	Interview and record review
Habte et al. (2021) [49]	SNNP	Case control	322	81	N/A	Interview and record review
Liyew et al. (2018) [50]	Addis Abeba	Case–control	864	216	N/A	Interview and record review
Dessalegn et al. (2020) [51]	Oromia	Case–control	321	80	N/A	Interview and record review
Kasahun et al. (2018) [31]	SNNP	Case–control	229	77	N/A	Interview and record review
Mekango et al. (2017) [32]	Tigray	Case control	308	103	N/A	Interview
Gaze Tenaw et al. (2021) [36]	Addis Abeba	Case control	432	108	N/A	Chart review

Key: N/A, Not applicable

### Prevalence of maternal near miss

**Fig. 2:**
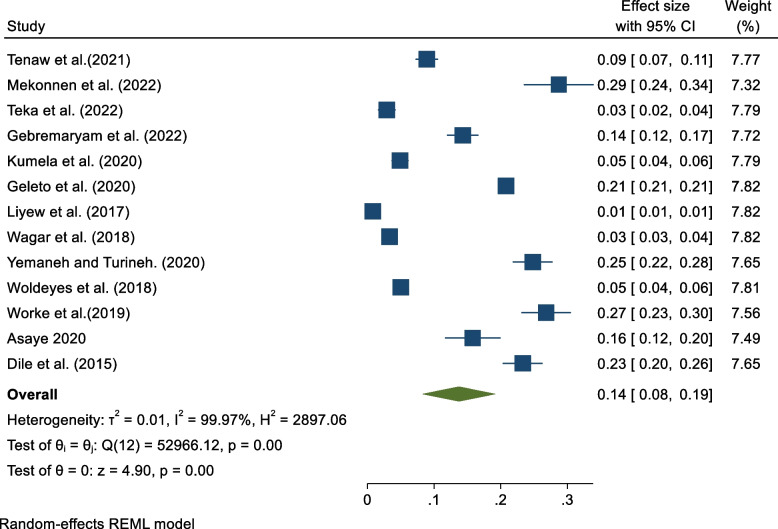
Forest plot indicating a pooled prevalence of maternal near misses in Ethiopia, 2023

The pooled prevalence of maternal near misses from 13 studies indicated 14% [95% CI: 8, 19]. Considerable heterogeneity was observed across the included studies [I^2^ = 99.98, *p* < 0.001] (Fig. 2).

### Subgroup analysis

**Fig. 3:**
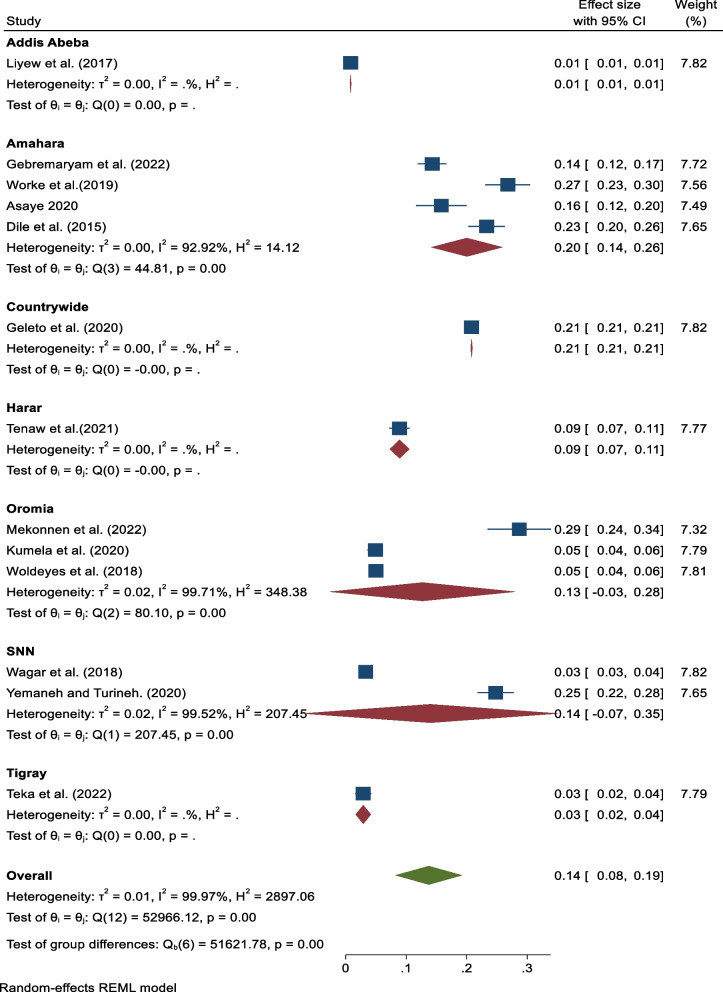
Subgroup analysis based on study area showing the pooled prevalence of maternal near miss in Ethiopia, 2023

Subgroup analysis based on the study setting (region) didn’t show any significant variation. Based on the subgroup analysis result, the highest prevalence (20%, 95% CI: 14, 26) I^2^ = 99.92%) was seen in the Amhara region, and the lowest prevalence (3%, 95% CI: 2, 4), I^2^ = . %) was seen in the Tigray region (Fig. 3).

**Fig. 4:**
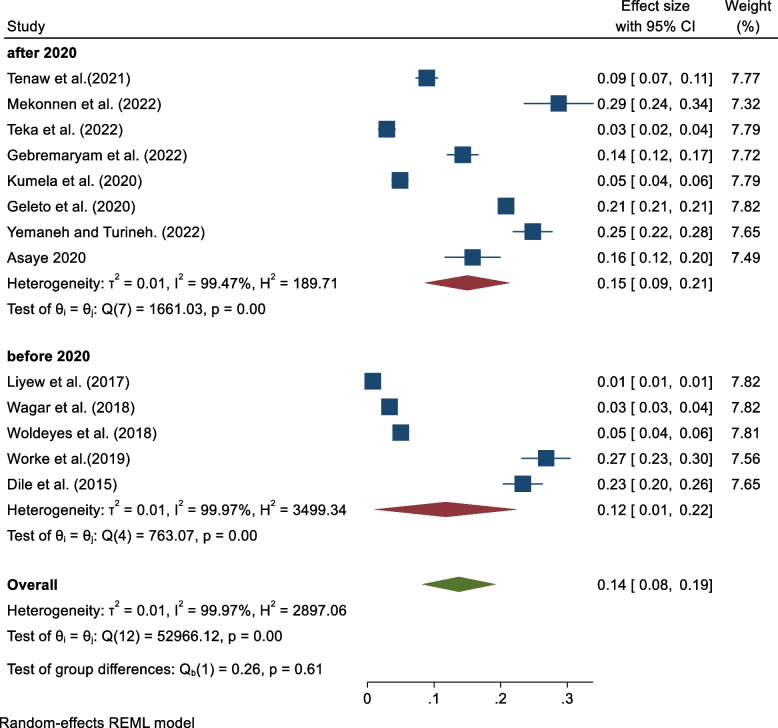
Subgroup analysis based on year of publication

There is no significant variation between studies published before and after 2020 (Fig. 4).

### Sensitivity analysis

The result of the sensitivity analysis done by dropping large and small sample sizes alternatively indicated there was no significant difference (Table 2).

**Table 2:** Sensitivity analysis to see the effect of sample size on outcome

Criteria	Initial prevalence	After analysis (%)	Heterogeneity
Dropping two small sample size	14% [95% CI: 8, 19]	12% [95% CI:6, 18]	I^2^ = 99.97%
After dropping three larger sample size	14% [95% CI: 8, 19]	15%[95% CI: 9, 21]	I^2^ = 99.16%
After dropping one larger sample size	14% [95% CI: 8, 19]	13% [95% CI: 7, 19]	I^2^ = 99.9%

### Factors associated with maternal near misses

**Fig. 5:**
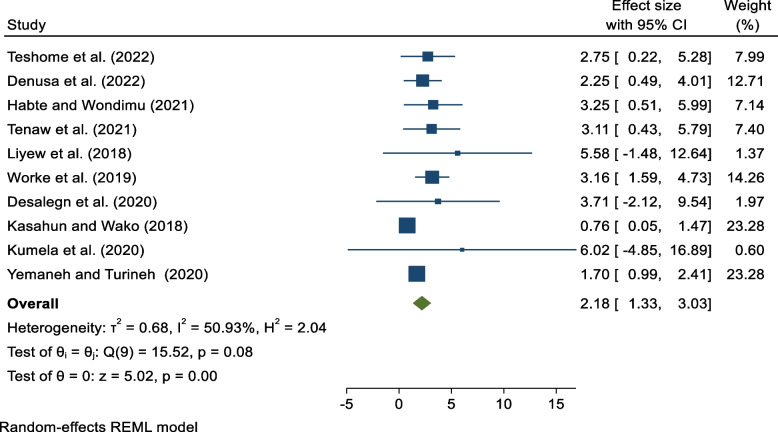
Association between lack of antenatal care and maternal near miss in Ethiopia 2023

Nine studies were included to assess the relationship between residence and MNM [31, 32, 36, 38, 43, 47, 49–51]. From the pooled estimate, there is no association between rural residence and maternal near misses [AOR 0.98, 95% CI, 0.66, 1.30]. Ten studies were included to evaluate the association between ANC and MNM [15, 31, 36, 43, 44, 47–51]. From pooled estimates, mothers who have no ANC were 2.18 times more likely to develop MNM when compared to those who have ANC [AOR = 2.18, 95% CI: 1.33, 3.03] (Fig. 5).

**Fig. 6:**
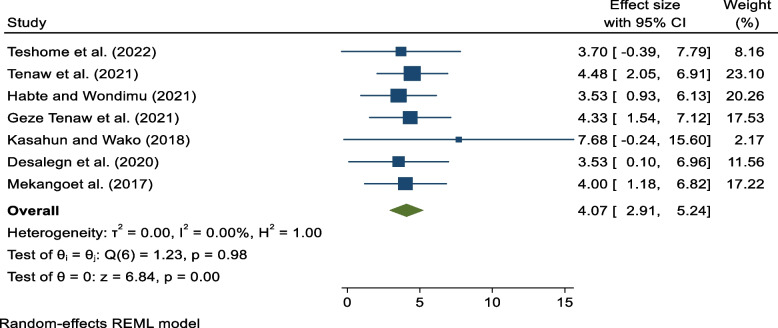
Association between history cesarean section and maternal near miss in Ethiopia 2023

The association between cesarean section (CS) and maternal near-miss was also assessed by 7 studies [31, 32, 36, 37, 47, 49, 51]. A pooled estimate revealed that a mother who has a history of cesarean section is 4.07 times more likely to develop MNM when compared to those who have no history of CS [AOR = 4.07, 95% CI: 2.91, 5.24]. There is no observed heterogeneity (I^2^ = 0) (Fig. 6).

Three studies were pooled to assess the association between anemia in index pregnancy and MNM [36, 37, 50]. Those who had anemia were 4.86 times more likely to develop MNM when compared to those who had no anemia [AOR = 4.86; 95% CI: 3.24, 6.47]. There is no heterogeneity in the study [I^2^ = 0.00%]. The effect of chronic medical disorders on MNM was assessed by a pooled estimate of five studies [32, 37, 47, 49, 51]. Those who have had a history of chronic medical disorders were 2.41 times more likely to develop MNM when compared to their counterparts [AOR = 2.41; 95% CI: 1.53, 3.29]. There is no variability among the included studies [I^2^ = 0.00%]. Four studies were included to evaluate the effect of educational level on MNM [32, 48, 49, 51]. The pooled estimate indicated that those who have no formal education were 2.10 times more likely to develop MNM when compared to the educated group [AOR = 2.10; 95% CI: 1.09, 3.10].

Additionally, factors like grand multiparty, residence, and a delay of 60 min were pooled to look at their effects. Even though those predictors were significantly associated in some studies, the pooled estimate fails to indicate a significant association.

## Discussion

The prevalence and factors associated with maternal near miss in Ethiopia were assessed and analyzed. From the 21 studies included in this systematic review and meta-analysis, 13 were used to compute the pooled prevalence, while 14 studies were used to assess factors associated with maternal near miss.

The pooled prevalence of maternal near miss from 13 studies (about 378,173 study participants) indicated 14% [95% CI: 8, 19]. This finding was greater than the global weighted pooled burden of maternal near misses [52], a study done in sub-Saharan Africa, Europe and Northern America [53], a study done in Tanzania [13], Latin America [54], Kenya [11], Harare, Namibia [55], Zimbabwe [56], South Africa [57], Eastern India [58] and Rwanda [59]. The reason for this variation could be the scope of the study, the year of publication, and the nature of the variation of MNM among high and low-income countries.

This finding is consistent with a systematic review and meta-analysis conducted in Ethiopia [19], a study conducted in Rwanda [60], Ghana [61], Indonesia and Western Pacific [62]. This could be because they belong to countries classified by the World Bank as developing countries and have high maternal morbidity and mortality rates.

The finding of this study was lower than those of the studies done in Uganda [14], and Uganda [63]. The difference between these findings may be because of variation between studies (systematic vs single study) and the difference in socio-demographics and health care systems of the study population.

In this meta-analysis, women who lack ANC follow-up were more likely to have maternal near miss. This finding was supported by a systematic review and meta-analysis (SRMA) in Ethiopia [19, 64]. This could be because lack of ANC is many things in obstetrics. It is a lack of screening, diagnosis and management, supplementation, health education [65] and a lack of birth preparedness complication readiness plan which resulted in poor pregnancy outcomes [66]. Also, those who have a history of cesarean section (CS) were 4.07 times more likely to have MNM. This finding was supported by a systematic review and meta-analysis study [67]. This is because the presence of a uterine scar could be one factor in the occurrence of complications like uterine rupture and hemorrhage [68]. Additionally, different complications can occur intraoperatively and can result in maternal near miss.

Another predictor was anemia, which increased 4.86 times the odds of having MNM among those who have anemia when compared to those who do not have anemia. Furthermore, those who had chronic medical disorders were 2.41 times more likely to have MNM when compared to their counterparts. This could be because medical disorders, including anemia, can be exacerbated by the physiologic nature of pregnancy and contribute to maternal MNM [69–72]. Educational status is another factor that affects maternal near miss. From the pooled estimate, participants who have no formal education were 2.10 times more likely to develop MNM when compared to the educated group. This could be because uneducated women may be less knowledgeable, limited awareness of their health and pregnancy danger signs, and are less likely to utilise maternity healthcare services [73–77].

This finding has considerable heterogeneity and should be interpreted with some limitations. The highest heterogeneity may be explained by the difference in study design or characteristics of the study population.

## Conclusion

The national prevalence of maternal near misses was still substantial. Lack of formal education, lack of ANC, history of cesarean section, anemia, and chronic medical disorders were among the predictors of maternal near miss. Antenatal care is found to be a special opportunity and area to intervene. More ANC contact as recommended by WHO should be practised. Avoiding primary cesarean section is recommended unless a clear indication is present. The country should give more emphasis on strategies for reducing MNM and its consequence, with the hope of improving women's health.

### Strengths and limitations of the study

The study provided pooled prevalence of maternal near misses which is more representative than a single study and is strong evidence. There is considerable heterogeneity across the study. This could be due to different study populations, study designs, data collection methods and sample sizes. Also, the funnel plot is asymmetric indicating publication bias, as any significant finding with maternal near-miss is more likely to be published. Therefore the interpretation of the finding should be cautious.

## Supplementary Information

**Additional file 1: Table.** Critical appraisal check list of quantitative studies of maternal near miss in Ethiopia 2023.

**Additional file 2: **Sample of searching engines.

## Data Availability

Additional data can be available from the corresponding author upon reasonable request.
