# Correction to: Prevalence and determinants of maternal near miss in Ethiopia: a systematic review and meta-analysis, 2015–2023

**DOI:** 10.1186/s12905-023-02565-z

**Published:** 2023-08-01

**Authors:** Abraham Negash, Addisu Sertsu, Dechasa Adare Mengistu, Aklilu Tamire, Adisu Birhanu Weldesenbet, Mesay Dechasa, Kabtamu Nigussie, Tilahun Bete, Elias Yadeta, Tegenu Balcha, Gebiso Roba Debele, Deribe Bekele Dechasa, Hamdi Fekredin, Habtamu Geremew, Jerman Dereje, Fikadu Tolesa, Magarsa Lami

**Affiliations:** 1 School of Nursing and Midwifery, College of Health and Medical Sciences, Haramaya University, Harar, Ethiopia; 2 School of Environmental Health, College of Health and Medical Sciences, Haramaya University, Harar, Ethiopia; 3 School of Public Health, College of Health and Medical Sciences, Haramaya University, Harar, Ethiopia; 4 School of Pharmacy, College of Health and Medical Sciences, Haramaya University, Harar, Ethiopia; 5 College of Health Sciences, Mattu University, Mattu, Ethiopia; 6 College of Health Sciences, Oda Bultum University, Chiro, Ethiopia; 7 College of Health Sciences, Salale University, Fitche, Ethiopia

Correction: BMC Women’s Health (2023) 23:1 10.1186/s12905-023-02523-9.

Following publication of the original article, in this article the author name ‘Tegenu Balcha’ was incorrectly written as ‘Taganu Balcha’

The original article has been corrected.

