# Supplementary material for: Prevalence and determinants of maternal near miss in Ethiopia: a systematic review and meta-analysis, 2015–2023
Source: BMC Womens Health. 2023 Jul 19;23:380. doi: 10.1186/s12905-023-02523-9 (PMC10357694; doi:10.1186/s12905-023-02523-9)
Supplement: Supplementary file 1 — Additional file 1: Table. Critical appraisal check list of quantitative studies of maternal near miss in Ethiopia 2023. [file 12905_2023_2523_MOESM1_ESM.docx]

**Appraisal**

Table: Critical appraisal check list of quantitative studies of maternal near miss in Ethiopia 2023. (1 =yes, 0=no/not mentioned); total score=9

| Studies | Q1 | Q2 | Q3 | Q4 | Q5 | Q6 | Q7 | Q8 | Q9 | Total score | Percent (%) |
| --- | --- | --- | --- | --- | --- | --- | --- | --- | --- | --- | --- |
| Tenaw et al. 2021 | Y | Y | Y | Y | Y | Y | Y | Y | Y | 9/9 | high |
| Mekonnen et al. 2021 | Y | Y | N | Y | Y | N | Y | Y | Y | 7/9 | moderate |
| Teka et al. 2022 | Y | N | Y | N | Y | Y | N | Y | Y | 6/8 | moderate |
| Gebremariam et al. 2022 | Y | Y | Y | Y | Y | Y | Y | Y | Y | 9/9 | high |
| Kumela et al. 2020 | Y | Y | N | Y | Y | Y | Y | N | U | 6/9 | moderate |
| Gelato et al. 2020 | Y | Y | Y | Y | Y | Y | Y | Y | Y | 9/9 | high |
| Liyew et al. 2017 | Y | Y | Y | Y | Y | Y | Y | Y | Y | 8/9 | high |
| Wagar et al. 2019 | Y | Y | Y | Y | Y | Y | Y | Y | Y | 8/9 | high |
| Yemaneh & Turineh 2020 | Y | U | Y | Y | Y | N | N | Y | Y | 7/9 | moderate |
| Woldeyes et al. 2019 | Y | Y | Y | Y | Y | Y | Y | N | Y | 8/9 | high |
| Worke et al. 2019 | Y | Y | Y | N | Y | Y | Y | Y | Y | 8/9 | high |
| Asaye 2020 | Y | Y | N | Y | Y | Y | Y | Y | Y | 8/9 | high |
| Dile et al. 2015 | Y | Y | Y | N | Y | Y | N | Y | Y | 7/9 | High |
| Teshome et al. 2022 | Y | Y | Y | Y | Y | Y | Y | Y | Y | 9/9 | High |
| Denusa et a. 2022 | Y | Y | Y | Y | Y | Y | Y | Y | Y | 9/9 | High |
| Habte and Wondimu 2021 | Y | Y | N | Y | Y | Y | Y | Y | Y | 8/9 | high |
| Liyew et al. 2018 | Y | Y | Y | Y | Y | Y | Y | Y | Y | 9/9 | high |
| Desalegn et al. 2020 | Y | Y | N | N | Y | Y | Y | Y | Y | 7/9 | moderate |
| Kasahun and Wako 2018 | Y | Y | Y | Y | Y | Y | Y | Y | Y | 9/9 | high |
| Makango et al. 2017 | Y | Y | Y | Y | N | Y | Y | Y | Y | 8/9 | high |
| Gaze Tenaw et al. 2021 | Y | Y | Y | Y | Y | Y | Y | Y | Y | 9/9 | high |

Notes:

Q1 - Was the sample frame appropriate to address the target population?

Q2 - Were study participants sampled in an appropriate way?

Q3 - Was the sample size adequate?

Q4 - Were the study subjects and the setting described in detail?

Q5 - Was the data analysis conducted with sufficient coverage of the identified sample?

Q6 - Were valid methods used for the identification of the condition?

Q7 - Was the condition measured in a standard, reliable way for all participants?

Q8 - Was there appropriate statistical analysis?

Q9 - Was the response rate adequate, and if not, was the low response rate managed appropriately?

Abbreviations: Y, yes; N, no; U, unclear
