# Supplementary material for: Prevalence and determinants of maternal near miss in Ethiopia: a systematic review and meta-analysis, 2015–2023
Source: BMC Womens Health. 2023 Jul 19;23:380. doi: 10.1186/s12905-023-02523-9 (PMC10357694; doi:10.1186/s12905-023-02523-9)
Supplement: Supplementary file 2 — Additional file 2: Sample of searching engines. [file 12905_2023_2523_MOESM2_ESM.docx]

**Sample of searching engineS**

**PUBMED searching engine**

Search query: ((((("epidemiology"[Subheading] OR "epidemiology"[All Fields] OR "prevalence"[All Fields] OR "prevalence"[MeSH Terms]) OR determinants[All Fields]) OR burden[All Fields]) AND ("mothers"[MeSH Terms] OR "mothers"[All Fields] OR "maternal"[All Fields]) AND "near miss"[All Fields]) AND (("mothers"[MeSH Terms] OR "mothers"[All Fields] OR "maternal"[All Fields]) AND near[All Fields] AND miss[All Fields])) AND Ethiopia[Title]

| **Year** | **Count** |
| --- | --- |
| 2023 | 1 |
| 2022 | 9 |
| 2021 | 9 |
| 2020 | 7 |
| 2019 | 2 |
| 2018 | 5 |
| 2017 | 2 |
| 2014 | 1 |

**Scopus searching engine**

**
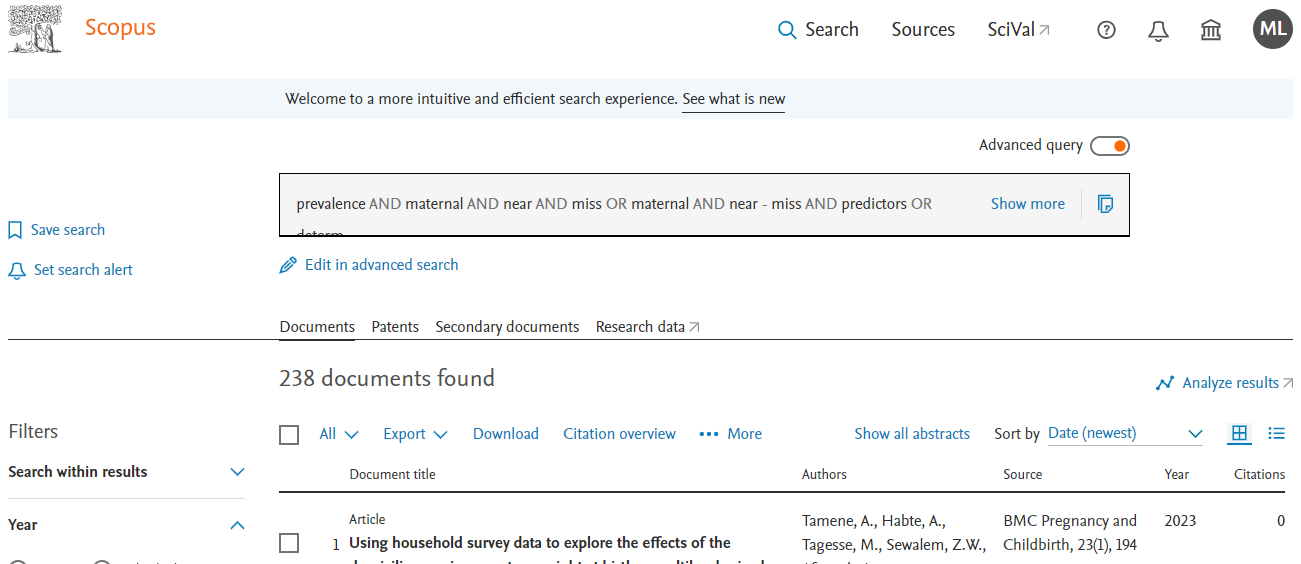
**

**CINAHIL searching engine**
